# Evaluation of the Impact of Advanced Glycation End-Products on Peripheral Neuropathy Outcomes in Type 2 Diabetes

**DOI:** 10.3390/biomedicines12112518

**Published:** 2024-11-04

**Authors:** Indumathi Singh, Tushar Issar, Ann M. Poynten, Kerry-Lee Milner, Arun V. Krishnan, Roshan Dhanapalaratnam

**Affiliations:** 1 Department of Neurology, Liverpool Hospital, Sydney, NSW 2170, Australia; 2 School of Clinical Medicine, University of New South WalesSydney, Kensington, NSW 2033, Australiaarun.krishnan@unsw.edu.au (A.V.K.); r.dhanapalaratnam@unsw.edu.au (R.D.); 3 Department of Endocrinology, Prince of Wales Hospital, Sydney, NSW 2031, Australia; ann.poynten@health.nsw.gov.au (A.M.P.);; 4 Department of Neurology, Prince of Wales Hospital, Sydney, NSW 2031, Australia

**Keywords:** advanced glycation end-products, diabetic peripheral neuropathy, skin autofluorescence and advanced glycation end-products

## Abstract

**Background**: Type 2 diabetes (T2DM) affects over 500 million people worldwide, and over 50% of this group experience the most common complication, diabetic peripheral neuropathy (DPN). The presence of advanced glycation end-products (AGEs) has been linked with the development of DPN. The present study assessed AGE levels in participants with type 2 diabetes and explored the hypothesis that there may be increased AGE levels in more severe DPN. **Methods**: A total of 124 participants with T2DM were consecutively recruited, and they underwent skin autofluorescence, clinical assessment for peripheral neuropathy, peripheral nerve ultrasound, nerve conduction studies, and axonal excitability assessment. **Results**: AGE accumulation showed weak but significant correlations with neuropathy severity and reduced nerve conduction function. However, after adjusting for confounding variables, a linear regression analysis did not reveal significant associations between the AGE levels and neuropathy outcomes. **Conclusions**: The present study suggests that the accumulation of AGE is not associated with the clinical, electrophysiological, and morphological measures of neuropathy in T2DM.

## 1. Introduction

Type 2 diabetes mellitus (T2DM) is a chronic metabolic disorder affecting over 500 million individuals worldwide, posing significant public health challenges. A common and debilitating complication of T2DM is diabetic peripheral neuropathy (DPN), which impacts more than 50% of diabetic patients and leads to progressive nerve injury, resulting in pain, a loss of sensation, and impaired motor function [1,2]. The multifactorial pathogenesis of DPN involves complex biochemical and physiological processes, including oxidative stress, inflammation, and vascular dysfunction [3,4].

Among the numerous factors implicated in the development of DPN, advanced glycation end-products (AGEs) have attracted considerable attention as potential contributors to the development of nerve dysfunction. AGEs are formed through the non-enzymatic glycation of proteins, lipids, and nucleic acids, a process exacerbated by chronic hyperglycemia [5]. These compounds accumulate in various tissues over time, altering their structure and function, and have been linked to diabetic complications such as retinopathy and nephropathy [6,7]. However, their role in the pathogenesis of DPN remains less well defined in the clinical context.

AGEs contribute to nerve damage in diabetic peripheral neuropathy (DPN) through multiple interrelated mechanisms. One key mechanism is the promotion of oxidative stress, where AGEs generate reactive oxygen species that lead to cellular damage, particularly in neuronal tissues [8]. This oxidative stress not only directly injures nerve cells but also exacerbates inflammation, further contributing to neuronal injury. AGEs are known to induce chronic inflammation by activating pro-inflammatory pathways, which perpetuate and amplify nerve damage over time. Additionally, AGEs impair normal cellular repair processes by modifying proteins through glycation, which disrupts cellular signaling pathways critical for maintaining nerve integrity and function. This glycation process leads to the formation of abnormal cross-links in extracellular matrix proteins, hindering normal cellular function and repair. These effects collectively contribute to the progressive degeneration of the peripheral nerves observed in DPN, resulting in symptoms such as pain, numbness, and motor dysfunction [9].

AGEs can be categorized as fluorescent or non-fluorescent. Fluorescent AGEs include glucose-derived AGEs (Glu-AGEs), fructose-derived AGEs (Fru-AGEs), methylglyoxal-derived AGEs (MGO-AGEs), and glyoxal-derived AGEs (GO-AGEs). The non-fluorescent AGEs are glyceraldehyde-derived AGEs (Glycer-AGEs), glycolaldehyde-derived AGEs (Glycol-AGEs), and 3-Deoxyglucosone-derived AGEs (3-DG-AGEs). Fluorescent AGEs can emit fluorescence when exposed to specific wavelengths of light, making them measurable through techniques like skin autofluorescence (SAF). SAF primarily assesses the accumulation of these fluorescent AGEs in the skin, serving as a non-invasive indicator of the overall AGE levels and related oxidative stress. Non-fluorescent AGEs, while significant in terms of their biological impact, are not typically detectable using SAF.

Beyond the established role in diabetic peripheral neuropathy, AGEs contribute significantly to the development of diabetic retinopathy and diabetic nephropathy. In retinopathy, AGEs induce retinal endothelial dysfunction, leading to increased vascular permeability, capillary occlusion, and subsequent progression of retinal damage. Similarly, in nephropathy, AGEs promote glomerular basement membrane thickening, mesangial expansion, and fibrosis, which contribute to the decline in kidney function and ultimately, diabetic kidney disease.

In terms of macrovascular complications, AGEs have been strongly implicated in the acceleration of atherosclerosis, a condition characterized by the thickening and hardening of arterial walls. AGEs induce endothelial cell dysfunction, reduce nitric oxide bioavailability, and promote vascular inflammation, all of which contribute to the development of coronary artery disease, stroke, and peripheral artery disease. These effects are exacerbated by the chronic hyperglycemic state present in diabetes, which fosters the accumulation of AGEs in vascular tissue.

Recent studies have demonstrated the utility of skin autofluorescence (SAF) as a method of detecting elevated AGE levels [10,11]. To better understand the associations between AGE levels and the severity of DPN, this study collected comprehensive data relating to peripheral nerve structure and function from participants with T2DM. AGE levels assessed using skin autofluorescence. This was correlated with clinical data, including age, sex, body mass index (BMI), waist circumference, duration of diabetes, HbA1c, triglycerides, LDL cholesterol, HDL cholesterol, total cholesterol, estimated glomerular filtration rate (eGFR), and the presence of chronic kidney disease.

## 2. Materials and Methods

A total of 124 participants with type 2 diabetes were consecutively recruited from the Diabetes Centre at Prince of Wales Hospital, Sydney. The participants underwent comprehensive assessments, including SAF measurement, clinical evaluation for peripheral neuropathy severity, peripheral nerve ultrasound, nerve conduction studies, and axonal excitability testing. Participants were excluded if they were younger than 18 years of age, were receiving glucagon-like peptide-1 receptor agonists [12], or had peripheral neuropathy due to alternate causes such as immune-mediated disease, nitrous oxide use, vitamin B12 deficiency, excess alcohol use, or exposure to neurotoxic medications such as chemotherapy [12].

Skin autofluorescence was measured using the AGE Reader (DMU00101, DiagnOptics Technologies, Groningen, The Netherlands) to assess fluorescent AGEs, such as pentosidine and other fluorescent compounds. This method specifically measures AGEs that exhibit autofluorescence, but it does not capture non-fluorescent AGEs. Fluorescent AGEs are a subset of advanced glycation end-products that emit light when exposed to specific wavelengths. Measurements were taken on the inner side of the forearm in a controlled environment with consistent ambient temperature (22–24 °C) and lighting conditions. Each participant underwent three consecutive measurements, and the mean SAF value was recorded.

A single trained neurologist conducted the clinical examination for peripheral neuropathy and completed the Total Neuropathy Score (TNS) and modified Toronto Clinical Neuropathy Score (mTCNS). The evaluation included a physical examination and a review of medical history focusing on peripheral neuropathy symptoms. The modified Toronto Clinical Neuropathy Scale includes six symptom scores and five examination values, all scored from 0 to 3 based on severity, with a maximum score of 33 [13]. The Total Neuropathy Score yields a maximum value of 32, scoring 8 sections from 0 to 4 each, including sensory and motor symptoms and signs, deep tendon reflexes, and sural and tibial nerve amplitudes [14]. Nerve conduction studies were conducted following standard protocols to assess the sural and tibial nerves (Natus, Middleton, WI, USA) [15].

High-resolution ultrasound was performed on the median and tibial nerves by a single ultrasonographer. Images were captured with a 10–18 MHz linear array transducer (MyLab™One, Esaote, Genoa, Italy). The musculoskeletal factory setting was used (acoustic power 100%, medium line density, dynamic range of 14, and persistence of 1) with constant gain and focus settings, and depth was varied as required to capture data. The probe was maintained at a 90-degree angle. The outcome measure of mean peripheral nerve cross-sectional area (CSA) was recorded for each participant after three free-hand traces of the inner margin of the endoneurium were obtained from each nerve. The median nerve CSA was tracked proximally from the wrist, and measurements were obtained at a site one-third of the length up the forearm in the participant’s dominant upper limb, away from any potential entrapment site [16]. The tibial nerve CSA was tracked proximally along with tibial vessels to a point 5 cm away from the medial malleolus, and measurement was recorded here away from any potential entrapment site [17].

Axonal excitability was undertaken on the median nerve and abductor pollicis brevis muscle in the participant’s dominant upper limb, using nonpolarizing surface electrodes (Ambu, Sydney, Australia), DS5 Isolated Bipolar Current Stimulator and the QtracW (Digitimer, London, UK), and the TROND protocol [18]. Skin temperature was maintained above 32 degrees Celsius. Stimulus–response curves were generated by using 1 ms pulses to achieve the highest CMAP amplitude, and then calculating 40% of the maximal response. The current required to achieve this target response was plotted for strength–duration, threshold electrotonus, and recovery cycle paradigms. The strength–duration relationship was tracked after using 0.2, 0.4, 0.8, and 1 ms duration stimuli. The strength–duration time constant (SDTC) was obtained using Weiss’ law to indirectly reflect the node of Ranvier persistent Na+ conductance [19]. Rheobase, the minimum current of any duration needed to elicit a peripheral nerve response, was also recorded. Threshold electrotonus, which provides insight into various nodal and internodal conductances, was tracked using depolarizing and hyperpolarizing conditioning currents at +40 and −40% of the control threshold, and percentage change in threshold was plotted at various 10 ms timepoints. For depolarizing threshold electrotonus, S2 accommodation, which is the period following peak reduction and threshold reduction returns to control threshold values, was also calculated. Hyperpolarizing threshold changes were recorded at 10–20 ms, 20–40 ms, and 90–100 ms timepoints. The recovery cycle was recorded by plotting threshold changes from 2 to 200 ms conditioning timepoints after the supramaximal stimulation of 1 ms duration. The parameters that were recorded were the relative refractory period, super excitability, and late sub excitability [20].

### 2.1. Ethical Considerations

The study protocol was reviewed and approved by eEthics review board, 2019/ETH04982 for South Eastern Sydney Local Health District Human Research Ethics Committee ensuring all the patient data were handled with strict confidentiality and in compliance with the ethical research standards.

### 2.2. Statistical Analysis

A single investigator blinded to all the participant data undertook data analysis using IBM SPSS Statistics version 26 (IBM Corp., Armonk, NY, USA). Normally distributed data are represented as the mean and standard deviation while non-normally distributed data, results are presented as the median and interquartile range (Q1–Q3). By convention, nerve excitability outcomes are presented as mean and standard error (SE). Correlation analyses were applied to determine the relationship between SAF and clinical measures as well as neuropathy outcomes. Pearson correlations were used for normally distributed data while Spearman correlations were used for non-normally distributed data. A multiple linear regression analysis was subsequently undertaken to model the relationship between SAF values and neuropathy outcomes. Neuropathy measures selected to be modeled were those determined to have a significant correlation with SAF in prior analyses. The model also included various confounding clinical variables, namely age, sex, BMI, HbA1c, diabetes duration, eGFR, and total cholesterol. To avoid multicollinearity, two or more similar clinical measures were not included, for example, BMI and waist circumference or total cholesterol and triglycerides. Various subsequent linear regression models were explored with a reduced subset of confounding clinical variables that had the largest contribution to the model, namely age, diabetes duration, and eGFR. A significance threshold of *p* < 0.05 was set for all the inferential testing to determine statistically significant differences.

## 3. Results

### 3.1. Participant Demographics and Clinical Characteristics

The demographic and clinical characteristics of the study cohort are summarized in Table 1. The study included 124 participants, with a male–female ratio of 77:57. The average age of the participants was 66 ± 13 years, reflecting an older cohort commonly affected by diabetic complications. The participants had a median diabetes duration of 144 months (IQR: 76–240), indicative of prolonged exposure to hyperglycemia, a key factor in the development of diabetic neuropathy and other complications.

The median body mass index (BMI) was 31.2 kg/m^2^ (28.4–36.6), with a median waist circumference of 111 cm (IQR: 100–117), highlighting the high prevalence of obesity within the cohort. Glycemic control, measured by HbA1c, had a median value of 7.9% (IQR: 7.2–9.0), with 72 participants (58%) receiving insulin therapy, indicating the moderate-to-poor control of blood glucose levels in a significant portion of the cohort.

Additionally, the median skin autofluorescence (SAF), a non-invasive measure of advanced glycation end-product (AGE) accumulation, was 3.3 arbitrary units (IQR: 2.9–3.9). This suggests substantial AGE deposition, which is commonly seen in patients with long-term diabetes. Metabolic parameters also reflected a range of cardiovascular risk factors. The lipid profile showed a median triglyceride level of 1.8 mmol/L (IQR: 1.3–2.8), LDL cholesterol of 1.6 mmol/L (IQR: 1.1–2.1), HDL cholesterol of 1.1 mmol/L (IQR: 0.9–1.3), and total cholesterol of 3.7 mmol/L (IQR: 3.3–4.6). Renal function was also assessed, with a median eGFR of 77 mL/min/1.73 m^2^ (IQR: 50–90), and 45 participants (36%) were classified as having chronic kidney disease (CKD). The median potassium level was 4.4 mmol/L (IQR: 4.1–4.6), which was within the normal range.

### 3.2. Peripheral Nerve Ultrasound and Nerve Conduction Data

The peripheral nerve ultrasound and nerve conduction data are summarized in Table 2. The peripheral nerve ultrasound revealed that the median nerve cross-sectional area (CSA) was 7.8 ± 1.7 mm^2^, and the tibial nerve CSA was 14.2 mm^2^ (IQR: 11.6–17.2), suggesting mild nerve enlargement consistent with diabetic neuropathy. The median nerve conduction studies showed a compound muscle action potential (CMAP) of 7.6 ± 2.7 mV and a latency of 7.2 ms (IQR: 6.6–8.0).

For the sural nerve, the sensory nerve action potential (SNAP) amplitude had a median value of 4.6 µV (IQR: 0–9.8), with a conduction velocity of 40.9 m/s (IQR: 0–50). The tibial nerve conduction results showed a median CMAP of 5.9 mV (IQR: 2.3–9.9) and a latency of 4.0 ms (IQR: 3.7–4.5). Neuropathy was also assessed using clinical measures: the cohort had a median Total Neuropathy Score (TNS) of 6 (IQR: 2–11) and a modified Toronto Clinical Neuropathy Score (mTCNS) of 4 (IQR: 2–11), indicating mild-to-moderate neuropathy in most participants.

#### 3.2.1. Correlation Between AGE, Clinical Measures, and Neuropathy Measures

The correlation between SAF and metabolic parameters is outlined in Table 3. SAF was positively correlated with diabetes duration (r = 0.338, *p* < 0.01), indicating that AGE accumulation increases with prolonged exposure to hyperglycemia. A positive correlation was also noted between SAF and potassium levels (r = 0.170, *p* < 0.05). In contrast, significant negative correlations were observed between SAF and several key metabolic measures, including BMI (r = −0.180, *p* < 0.05), triglycerides (r = −0.265, *p* < 0.01), total cholesterol (r = −0.277, *p* < 0.01), and eGFR (r = −0.393, *p* < 0.01). These findings suggest that while AGE accumulation is closely associated with declining kidney function and long-term diabetes, it may not directly reflect lipid profiles or body mass. No significant correlations were found between SAF and waist circumference, HbA1c, LDL cholesterol, or HDL cholesterol, indicating that SAF may not fully capture the complexity of metabolic health in this cohort.

#### 3.2.2. Linear Regression Modeling Between AGE and Neuropathy Outcomes

The results of the linear regression analysis, presented in Table 4, demonstrated that none of the neuropathy outcomes were significantly associated with the AGE levels after controlling for the confounding factors, including age, sex, diabetes duration, HbA1c, eGFR, total cholesterol, and BMI (indicated by the asterisk). Although weak associations were noted between the AGE levels and tibial nerve CSA (β = 0.149), median nerve CMAP (β = −0.133), sural nerve SNAP (β = −0.102), sural nerve velocity (β = −0.117), and tibial nerve CMAP (β = −0.083), these relationships did not reach statistical significance. Similarly, the clinical neuropathy scores, TNS (β = 0.135) and mTCNS (β = 0.068), exhibited weak association with the AGE levels. A subsequent modeling analysis was undertaken with reduced subsets limited to the confounding variables with the strongest influence in the model, namely age, diabetes duration and eGFR; however, no significant association was found between AGE and neuropathy outcomes. Overall, the regression analysis suggests that while AGE accumulation contributes to systemic metabolic dysfunction, it does not appear to play a significant role in the electrophysiological or structural changes associated with diabetic neuropathy in this cohort.

Table 1: Demographic and clinical characteristics of the 124 study participants. The data include age, sex distribution, BMI, waist circumference, duration of diabetes, HbA1c levels (both in percentage and IFCC units), lipid profile, renal function (eGFR), and the prevalence of chronic kidney disease (CKD) within the cohort. Normally distributed data are displayed as mean ± SD, while non-normally distributed data are written as median (Q1–Q3).

Table 2: Peripheral nerve ultrasound and nerve conduction study data for the cohort. Cross-sectional area (CSA) and compound muscle action potentials (CMAPs) and nerve conduction latencies of the median and tibial nerves are displayed along with sural nerve sensory action potential (SNAP) amplitude and velocity. Additionally, the Total Neuropathy Score (TNS) and modified Toronto Clinical Neuropathy Score (mTCNS) are included as the measures of neuropathy severity. Normally distributed data are displayed as mean ± SD, while non-normally distributed data are written as median (Q1–Q3).

Table 3: Correlation coefficients (r) between skin autofluorescence, a measure of advanced glycation end-products (AGEs), and various clinical parameters including BMI, waist circumference, diabetes duration, HbA1c, lipid profile, eGFR, and potassium levels. Statistically significant correlations are denoted by * *p* < 0.05 and ** *p* < 0.01. Normally distributed data are displayed as mean ± SD, while non-normally distributed data are written as median (Q1–Q3).

Table 4: Standardized β-coefficients from linear regression models examining the relationship between AGE levels and neuropathy outcomes, including nerve cross-sectional area (CSA), compound muscle action potential (CMAP), sensory nerve action potential (SNAP), and clinical neuropathy scores (TNS and mTCNS). The models control for the confounding factors age, sex, diabetes duration, HbA1c, eGFR, total cholesterol, and BMI. BMI was selected over waist circumference as it was significantly associated with AGE as demonstrated in Table 3. Abbreviations: CSA, cross-sectional area; CMAP, compound muscle action potential; SNAP, sensory nerve action potential; TNS, Total Neuropathy Score; mTCNS, modified Toronto Clinical Neuropathy Score.

## 4. Discussion

This study assessed the relationship between AGEs and the severity of DPN in a cohort of 124 individuals with T2DM. While AGEs are established contributors to various diabetic complications, our findings indicate that the accumulation of AGEs is not a robust predictor of DPN severity when compared to the direct assessments of peripheral nerve structure and function.

Notably, significant correlations were observed between the AGE levels and several metabolic parameters, including BMI, triglycerides, total cholesterol, and eGFR, corroborating prior research that links metabolic disturbances with increased AGE accumulation [21,22,23]. The positive association between the AGE levels and diabetes duration further substantiates the hypothesis that prolonged hyperglycemia fosters progressive AGE deposition in tissues [4,24]. While initial correlations suggested relationships between elevated AGE levels and neuropathy-specific outcomes, these associations did not remain significant after controlling for confounding variables. These findings imply that despite their role in metabolic dysfunction, AGEs do not directly modulate the severity of neuropathy, as measured by these objective parameters [25,26].

The absence of a strong relationship between AGE levels and peripheral nerve function underscores the multifactorial nature of DPN pathogenesis. It suggests that other pathogenic mechanisms, including oxidative stress, chronic inflammation, and microvascular impairment, may exert more pronounced effects on DPN development and progression than AGE accumulation alone [21,26,27]. In addition to AGE accumulation, the polyol pathway contributes to diabetic neuropathy through the accumulation of sorbitol. Under the conditions of hyperglycemia, excess glucose is converted to sorbitol, which accumulates in nerve cells, causing osmotic stress and disrupting cellular function. Sorbitol also depletes NADPH, weakening antioxidant defenses and increasing oxidative stress while reducing myo-inositol levels, which impairs nerve conduction. Unlike AGEs, which cause long-term tissue damage via protein cross-linking and inflammation, sorbitol disrupts the metabolic balance within nerve cells, leading to more acute cellular dysfunction. Both pathways contribute to oxidative stress but through distinct mechanisms: sorbitol via metabolic disruption and AGEs via chronic structural changes. Recognizing the dual impact of sorbitol and AGEs reiterates the need for comprehensive approaches to managing diabetic neuropathy. This emphasizes the importance of employing comprehensive peripheral nerve assessments, such as nerve conduction studies and axonal excitability tests, which are sensitive to subtle alterations in nerve health that may not be captured by AGE measurements alone.

Our findings differ from a few earlier studies that reported stronger associations between AGE levels and neuropathy outcomes. Additionally, studies have linked AGEs to microvascular dysfunction, which further exacerbates nerve damage by reducing blood flow to the peripheral nerves. Several studies have reported a correlation between advanced glycation end-products (AGEs) and diabetic peripheral neuropathy (DPN). One study highlighted that while AGE accumulation is linked to neuropathy, SAF lacks the specificity to be considered a precise diagnostic tool [28]. Another study found a correlation between AGE levels and type 2 DPN but concluded that AGEs alone do not provide sufficient diagnostic sensitivity [10]. A third study noted inconsistencies in the diagnostic reliability of SAF [11], and further research suggested that although SAF may be useful in screening for neuropathy, its poor sensitivity prevents it from being a standalone diagnostic tool [29]. Consistent with these findings, our study did not observe significant correlations between AGE levels and neuropathy outcomes after adjusting for confounding factors. Despite these findings, research has produced mixed results, with some studies showing weak or inconsistent correlations between AGEs and neuropathy severity, suggesting that the relationship between AGEs and DPN may involve additional factors [27,30].

These discrepancies could be attributed to variations in study design, population characteristics, or methodological differences in AGE measurement, such as disparities in skin autofluorescence devices and calibration standards. Furthermore, our use of advanced diagnostic modalities, including nerve excitability testing and high-resolution nerve ultrasound, alongside a heterogeneous patient cohort, may have provided a more nuanced understanding of neuropathy, potentially explaining the weaker associations observed. The specific characteristics of our study population, including prolonged diabetes duration, better glycemic control, and varied comorbidities, may have influenced the results. It is plausible that other mechanisms of nerve damage, such as oxidative stress and vascular insufficiency, overshadowed the impact of AGE accumulation in this cohort [21].

The current literature on AGEs and diabetic neuropathy is fragmented, with various studies exploring different aspects of AGE formation, accumulation, and their impacts on diabetic complications. This makes it challenging to form a cohesive understanding that links AGEs directly to the pathogenesis of neuropathy. There is a requirement for longitudinal studies and meta-analyses that combine data from multiple sources to create a more comprehensive understanding. Future research should focus on exploring the various pathways that link AGEs with nerve damage in diabetes, potentially through combined biochemical, histological, and clinical studies. Our results underscore the complex and multifactorial nature of DPN, wherein AGEs represent just one element of the broader pathogenic landscape [25]. Our rigorous adjustment for potential confounders, including eGFR, age, sex, duration of diabetes, HbA1c, and BMI, further suggests that while AGEs play a role in DPN, they do not fully account for its severity, highlighting the necessity of considering multiple factors in the pathogenesis of DPN. It is important to consider potential confounders such as eGFR, age, sex, duration of diabetes, HbA1c, and waist circumference in exploring the multifactorial pathogenesis of diabetic peripheral neuropathy (DPN). Each of these variables can significantly influence neuropathy progression, providing important context to better understand the complex mechanisms at play beyond the role of AGEs. eGFR serves as a marker of renal function, and impaired kidney function may exacerbate neuropathy by promoting metabolic disturbances and AGE accumulation. The duration of diabetes is well established as a predictor of neuropathy, with longer disease duration linked to chronic hyperglycemia and sustained metabolic damage, including but not limited to AGE-related mechanisms. Moreover, HbA1c reflects long-term glycemic control, and its association with DPN is well documented, as persistent hyperglycemia drives not only AGE formation but also other pathological processes such as oxidative stress and inflammation. The observed association between higher AGE levels and diabetic kidney disease, but not neuropathy severity, suggests that AGEs may be more pertinent to other diabetes-related complications, such as nephropathy [2,4]. This finding is particularly relevant for clinical practice, as it emphasizes the need for targeted diagnostic and therapeutic strategies tailored to the specific complications associated with diabetes.

Our study used skin autofluorescence (SAF) to measure fluorescent AGEs. However, SAF does not capture non-fluorescent AGEs. This limits our assessment to a subset of total AGE accumulation. To fully understand the impact of all AGE types, methods like serum or tissue biopsies would provide a broader measure. While SAF is a useful non-invasive tool, its limitations in detecting non-fluorescent AGEs should be considered when interpreting the results.

Non-fluorescent AGEs have been shown to play significant roles in tissue damage and metabolic dysfunction. A more comprehensive analysis that includes these non-fluorescent AGEs would provide a fuller picture of AGE-related pathophysiology in diabetic neuropathy. Further studies should consider including mass spectrometry or high-performance liquid chromatography (HPLC) methods for the precise quantification of a range of AGEs. This would enable a better understanding of how specific AGE subtypes correlate with neuropathy severity and progression. Studies in diabetic patients have shown that SAF, which measures fluorescent AGEs, is positively correlated with blood AGEs like pentosidine and carboxymethyl-lysine (CML). However, this correlation is not always consistent due to differences in how AGEs accumulate in the skin versus blood. In contrast, evidence for a correlation between skin AGEs and AGEs in peripheral nerve cells is limited. While AGEs are known to accumulate in nerves and contribute to diabetic neuropathy, the direct measurement of AGEs in nerve cells is not routinely performed. Therefore, skin AGE levels may not fully reflect AGE accumulation in the peripheral nerves. SAF may not be the best way to assess the relationship, given that we are comparing a cutaneous marker to a peripheral nerve endpoint.

AGE concentrations can also be measured in more stable biological fluids, such as exosomes. Exosomes, as extracellular vesicles, can carry AGE-modified proteins and reflect changes in cellular metabolism. They provide a stable medium that could more accurately reflect systemic AGE levels compared to skin measurements. Assessing AGEs in exosomes or other stable fluids like plasma or serum could improve the sensitivity and reliability of AGE measurements. Such an approach would also allow for longitudinal assessments, helping to track changes in AGE levels over time in response to therapeutic interventions or disease progression. To address this limitation, future studies could employ more precise methods such as serum assays, tissue biopsies, or advanced techniques like mass spectrometry. These methods could provide better insights into the specific AGE subtypes that might be more closely related to nerve damage in T2DM.

## 5. Conclusions

In summary, while AGEs are critical markers of metabolic stress in diabetes, our study indicates that they are not reliable predictors of DPN severity. Our results are within the broader context of diabetic neuropathy stressing that the lack of significant associations in our study should not be viewed as conclusive evidence against any role of AGEs in neuropathy. Instead, it emphasizes the multifactorial nature of diabetic neuropathy, which involves various interacting mechanisms beyond AGE accumulation alone. These findings highlight the importance of adopting a multifaceted approach that integrates direct nerve assessments to accurately evaluate and manage neuropathy. Shifting the focus to a broader understanding of the pathophysiological mechanisms underlying DPN may lead to more effective strategies for improving patient outcomes [4,24,25,26].

## Figures and Tables

**Table 1:** Patient demographics and clinical characteristics.

Parameter	Value
N	124
Age (years)	66 ± 13
Sex (Male: Female)	77:57:00
Skin autofluorescence	3.3 (2.9–3.9)
BMI (kg/m^2^)	31.2 (28.4–36.6)
Waist circumference (cm)	111 (100–117)
Diabetes duration (months)	144 (76–240)
HbA1c (%)	7.9 (7.2–9.0)
HbA1c (IFCC units)	62.8 (55.2–74.9)
Triglycerides (mmol/L)	1.8 (1.3–2.8)
LDL (mmol/L)	1.6 (1.1–2.1)
HDL (mmol/L)	1.1 (0.9–1.3)
Total cholesterol (mmol/L)	3.7 (3.3–4.6)
On insulin (Yes/No)	72:62
eGFR (ml/min/1.73m^2^)	77 (50–90)
CKD (Yes/No)	45:89
Potassium (mmol/L)	4.4 (4.1–4.6)

**Table 2:** Peripheral nerve ultrasound and nerve conduction data.

Parameter	Value
Median CSA (mm^2^)	7.8 ± 1.7
Tibial CSA (mm^2^)	14.2 (11.6–17.2)
Median CMAP (mV)	7.6 ± 2.7
Median latency (ms)	7.2 (6.6–8.0)
Sural SNAP (µV)	4.6 (0–9.8)
Sural velocity (m/s)	40.9 (0–50)
Tibial CMAP (mV)	5.9 (2.3–9.9)
Tibial latency (ms)	4.0 (3.7–4.5)
TNS	6 (2–11)
mTCNS	4 (2–11)

**Table 3:** Correlation between AGE, clinical measures, and neuropathy measures.

Clinical Parameter	Correlation with Skin Autofluorescence (r)
Age (years)	0.422 **
BMI	−0.180 *
Waist Circumference	−0.146
Diabetes Duration	0.338 **
HbA1c	0.095
Triglycerides	−0.265 **
LDL Cholesterol	−0.079
HDL Cholesterol	0.113
Total Cholesterol	−0.277 **
eGFR	−0.393 **
Potassium (K^+^)	0.170 *
Median CSA (mm^2^)	0.064
Tibial CSA (mm^2^)	0.187 *
Median CMAP (mV)	−0.302 **
Sural SNAP (µV)	−0.267 **
Sural Velocity (ms)	−0.284 **
Tibial CMAP (mV)	−0.266 **
Tibial Latency (ms)	0.102
TNS	0.237 **
mTCNS	0.184 *

Statistically significant correlations are denoted by * *p* < 0.05 and ** *p* < 0.01.

**Table 4:** Linear regression modeling between AGE and neuropathy outcomes.

Parameter	β-Coefficient
Tibial Nerve CSA (mm^2^)	0.149
Median Nerve CMAP (mV)	−0.133
Sural Nerve SNAP (µV)	−0.102
Sural Nerve Velocity (ms^−1^)	−0.117
Tibial Nerve CMAP (mV)	−0.083
Tibial Nerve Latency (ms)	0.102
Total Neuropathy Score (TNS)	0.135
Modified Toronto Clinical Neuropathy Score (mTCNS)	0.068

## Data Availability

The original contributions presented in the study are included in the article, further inquiries can be directed to the corresponding author.

## References

[1] Pop-Busui R., Boulton A.J., Feldman E.L., Bril V., Freeman R., Malik R.A., Sosenko J.M., Ziegler D. (2016). Diabetic neuropathy: A position statement by the American Diabetes Association. Diabetes Care.

[2] Tesfaye S., Boulton A.J.M., Dyck P.J., Freeman R., Horowitz M., Kempler P., Lauria G., Malik R.A., Spallone V., Vinik A. (2010). Diabetic neuropathies: Update on definitions, diagnostic criteria, estimation of severity, and treatments. Diabetes Care.

[3] Vincent A.M., Russell J.W., Low P., Feldman E.L. (2004). Oxidative stress in the pathogenesis of diabetic neuropathy. Endocr. Rev..

[4] Callaghan B.C., Cheng H.T., Stables C.L., Smith A.L., Feldman E.L. (2012). Diabetic neuropathy: Clinical manifestations and current treatments. Lancet Neurol..

[5] Brownlee M. (2001). Biochemistry and molecular cell biology of diabetic complications. Nature.

[6] Stitt A.W., Jenkins A.J., E Cooper M. (2002). Advanced glycation end products and diabetic complications. Expert. Opin. Investig. Drugs.

[7] Vlassara H., Uribarri J. (2014). Advanced glycation end products (age) and diabetes: Cause, effect, or both?. Curr. Diabetes Rep..

[8] Yan S.F., Ramasamy R., Schmidt A.M. (2008). Mechanisms of Disease: Advanced Glycation End-Products and Their Receptor in Inflammation and Diabetes Complications. Nat. Clin. Pract. Endocrinol. Metab..

[9] Goldin A., Beckman J.A., Schmidt A.M., Creager M.A. (2006). Advanced glycation end products: Sparking the development of diabetic vascular injury. Circulation.

[10] Zhao X.-W., Yue W.-X., Zhang S.-W., Chen Q. (2022). Correlation between the accumulation of skin glycosylation end products and the development of type 2 diabetic peripheral neuropathy. BMC Endocr. Disord..

[11] Papachristou S., Pafili K., Trypsianis G., Papazoglou D., Vadikolias K., Papanas N. (2022). Skin advanced glycation end products as a screening tool of neuropathy in type 2 diabetes mellitus. J. Diabetes Complicat..

[12] Dhanapalaratnam R., Issar T., Lee A.T.K., Poynten A.M., Milner K.-L., Kwai N.C.G., Krishnan A.V. (2024). Glucagon-like peptide-1 receptor agonists reverse nerve morphological abnormalities in diabetic peripheral neuropathy. Diabetologia.

[13] Bril V., Tomioka S., Buchanan R.A., Perkins B.A., the mTCNS Study Group (2009). Reliability and validity of the modified Toronto Clinical Neuropathy Score in diabetic sensorimotor polyneuropathy. Diabet. Med..

[14] Cornblath D., Chaudhry V., Carter K., Lee D., Seysedadr M., Miernicki M., Joh T. (1999). Total neuropathy score. Neurology.

[15] Burke D., Skuse N.F., Lethlean A.K. (1974). Sensory conduction of the sural nerve in polyneuropathy. J. Neurol. Neurosurg. Psychiatry.

[16] Won S.J., Kim B., Park K.S., Yoon J.S., Choi H. (2013). Reference values for nerve ultrasonography in the upper extremity. Muscle Nerve.

[17] Fisse A.L., Katsanos A.H., Gold R., Krogias C., Pitarokoili K. (2021). Cross-sectional area reference values for peripheral nerve ultrasound in adults: A systematic review and meta-analysis—Part II: Lower extremity nerves. Eur. J. Neurol..

[18] Burke D., Kiernan M.C., Bostock H. (2001). Excitability of human axons. Clin. Neurophysiol..

[19] Dhanapalaratnam R., Issar T., Poynten A.M., Milner K.-L., Kwai N.C., Krishnan A.V. (2024). Progression of axonal excitability abnormalities with increasing clinical severity of diabetic peripheral neuropathy. Clin. Neurophysiol..

[20] Kiernan M.C., Bostock H., Park S.B., Kaji R., Krarup C., Krishnan A.V., Kuwabara S., Lin C.S.-Y., Misawa S., Moldovan M. (2020). Measurement of axonal excitability: Consensus guidelines. Clin. Neurophysiol..

[21] Giacco F., Brownlee M. (2010). Oxidative stress and diabetic complications. Circ. Res..

[22] Ceriello A., Ihnat M.A., Thorpe J.E. (2009). The “metabolic memory”: Is more than just tight glucose control necessary to prevent diabetic complications?. J. Clin. Endocrinol. Metab..

[23] Gerrits E.G., Lutgers H.L., Kleefstra N., Graaff R., Groenier K.H., Smit A.J., Gans R.O., Bilo H.J. (2008). Skin autofluorescence: A tool to identify type 2 diabetic patients at risk for developing microvascular complications. Diabetes Care.

[24] Vlassara H., Striker G.E. (2013). Advanced glycation endproducts in diabetes and diabetic complications. Endocrinol. Metab. Clin. N. Am..

[25] Selvarajah D., Kar D., Khunti K., Davies M.J., Scott A.R., Walker J., Tesfaye S. (2019). Diabetic peripheral neuropathy: Advances in diagnosis and strategies for screening and early intervention. Lancet Diabetes Endocrinol..

[26] Pop-Busui R., Ang L., Holmes C., Gallagher K., Feldman E.L. (2016). Inflammation as a therapeutic target for diabetic neuropathies. Curr. Diab. Rep..

[27] Davies J.L., Clark V.M., Litchy W.J., Dyck P.J.B., Klein C.J., Rizza R.A., Pach J.M., Klein R., Larson T.S., Melton L.J. (2006). Modeling chronic glycemic exposure variables as correlates and predictors of microvascular complications of diabetes. Diabetes Care.

[28] Trofin D., Salmen B.-M., Salmen T., Trofin D.M., Reurean-Pintilei D. (2023). Advancing the Diagnosis of Diabetic Neuropathies: Electrodiagnostic and Skin Autofluorescence Methods. J. Pers. Med..

[29] Papachristou S., Pafili K., Papanas N. (2021). Skin AGEs and Diabetic Neuropathy. BMC Endocr. Disord..

[30] Vincent A.M., Callaghan B.C., Smith A.L., Feldman E.L. (2011). Diabetic neuropathy: Cellular mechanisms as therapeutic targets. Nat. Rev. Neurol..

